# Anaemia and its functional consequences in cancer patients: current challenges in management and prospects for improving therapy

**DOI:** 10.1054/bjoc.2001.1750

**Published:** 2001-04

**Authors:** G D Demetri

**Affiliations:** Center for Sarcoma and Bone Oncology, Dana-Farber Cancer Institute and Harvard Medical School, 44 Binney Street, Boston, MA, 02115, USA

**Keywords:** anaemia, cancer, chemotherapy, radiotherapy, recombinant human erythropoietin, darbepoetin alfa

## Abstract

Anaemia is a common occurrence in patients with cancer and contributes to the clinical symptomatology and reduced quality of life (QOL) seen in cancer patients. Many aspects of reduced QOL, including fatigue, are known to be associated with suboptimally low levels of haemoglobin. Even mild-to-moderate anaemia adversely affects patient-reported QOL parameters. Red blood cell transfusions are associated with many real and perceived risks, inconveniences, costs, and only temporary benefits. Recombinant human erythropoietin (rHuEPO) is an effective therapy to increase haemoglobin values in over half of anaemic cancer patients receiving concurrent chemotherapy. These increased haemoglobin values are closely correlated with improvements in QOL. Despite these objectively defined benefits, less than 50% of anaemic patients undergoing cytotoxic chemotherapy receive rHuEPO, in contrast to patients with chronic renal failure on dialysis, where anaemia is universally and aggressively treated to more optimal haemoglobin values. However, there are several barriers that may limit more widespread use of rHuEPO. These include inconvenience associated with frequent dosing; failure of a large proportion (40 to 50%) of patients to respond; relatively slow time to response; absence of reliable early indicators of response; and current lack of rigorous pharmacoeconomic data demonstrating cost-effectiveness. Darbepoetin alfa is a novel erythropoiesis stimulating protein (NESP) that is biochemically distinct from rHuEPO, and which has been proven to stimulate red blood cell production. The molecule has a 3-fold longer half-life and increased biological activity that will allow less frequent dosing, facilitating improved management of the anaemia of cancer. With this new option for therapy, further avenues of investigation should lead to renewed interest in the clinical benefits of optimal haemoglobin levels for patients with cancer. © 2001 Cance Cancer Research Campaign


*British Journal of Cancer* (2001) **84** (Supplement 1), 31–37
© 2001 Cancer Research Campaign
doi: 10.1054/ bjoc.2001.1750, available online at http://www.idealibrary.com on IDEAL®

### Anaemia and its functional consequences in cancer patients: current challenges in management and prospects for improving therapy

**GD Demetri**

Center for Sarcoma and Bone Oncology, Dana-Farber Cancer Institute and Harvard Medical School, 44 Binney Street, Boston, MA 02115, USA

**Summary** Anaemia is a common occurrence in patients with cancer and contributes to the clinical symptomatology and reduced quality of life (QOL) seen in cancer patients. Many aspects of reduced QOL, including fatigue, are known to be associated with suboptimally low levels of haemoglobin. Even mild-to-moderate anaemia adversely affects patient-reported QOL parameters. Red blood cell transfusions are associated with many real and perceived risks, inconveniences, costs, and only temporary benefits. Recombinant human erythropoietin (rHuEPO) is an effective therapy to increase haemoglobin values in over half of anaemic cancer patients receiving concurrent chemotherapy. These increased haemoglobin values are closely correlated with improvements in QOL. Despite these objectively defined benefits, less than 50% of anaemic patients undergoing cytotoxic chemotherapy receive rHuEPO, in contrast to patients with chronic renal failure on dialysis, where anaemia is universally and aggressively treated to more optimal haemoglobin values. However, there are several barriers that may limit more widespread use of rHuEPO. These include inconvenience associated with frequent dosing; failure of a large proportion (40 to 50%) of patients to respond; relatively slow time to response; absence of reliable early indicators of response; and current lack of rigorous pharmacoeconomic data demonstrating cost-effectiveness. Darbepoetin alfa is a novel erythropoiesis stimulating protein (NESP) that is biochemically distinct from rHuEPO, and which has been proven to stimulate red blood cell production. The molecule has a 3-fold longer half-life and increased biological activity that will allow less frequent dosing, facilitating improved management of the anaemia of cancer. With this new option for therapy, further avenues of investigation should lead to renewed interest in the clinical benefits of optimal haemoglobin levels for patients with cancer. © 2001 Cancer Research Campaign

**Keywords**: anaemia; cancer; chemotherapy; radiotherapy; recombinant human erythropoietin; darbepoetin alfa

In patients with cancer, particularly those receiving cytotoxic chemotherapy, anaemia is a common occurrence (Groopman and Itri, 1999). The condition can have a profound effect on many aspects of quality of life (QOL), such as exercise capacity, fatigue and mood (Cella, 1997). However, studies indicate that anaemia and its most frequent manifestation, fatigue, are under-recognized and under-treated (Vogelzang et al, 1997; Lawless et al, 2000). Several factors may contribute to seemingly suboptimal management of anaemia in cancer patients. These reasons relate to lack of awareness among physicians, inadequacies of current treatments, and the existence of several key outstanding issues regarding the general management of cancer-related anaemia. This review first summarizes the evidence showing the significance and benefits of treating anaemia in cancer patients and discusses the key factors contributing to the apparent under-treatment of anaemia.

### INCIDENCE OF CANCER-RELATED ANAEMIA

Anaemia may develop as a result of the malignant disease process itself; from bleeding, nutritional deficiencies, bone marrow damage, tumour infiltration of the bone marrow, or immunologic impairment of erythropoietic response. However, it probably occurs most often by iatrogenic means, as a consequence of myelosuppressive chemotherapy or radiotherapy (Beguin, 1996).

Several researchers have measured the incidence of anaemia in cancer patients (Coiffier, 1998; Dalton et al, 1998; Groopman and Itri, 1999). In a broad review of published clinical trials, Groopman and Itri (1999) found that the incidence of Grade 1 to 2 and Grade 3 to 4 anaemia after chemotherapy can be as high as 100% and 80%, respectively. The incidence of anaemia varies depending on tumour type and chemotherapy regimen. For example, the combination of cisplatin and etoposide, one of the most widely used regimens for advanced small-cell lung cancer, produced Grade 3 or 4 anaemia in 16 to 55% of patients. However, the combination of 5-fluorouracil and leucovorin in advanced colorectal cancer produced Grade 3 or 4 anaemia in only 2 to 5% of patients.

Chemotherapy undoubtedly contributes to the development of anaemia, and this incidence increases over the course of therapy (Coiffier, 1998; Dalton et al, 1998). Coiffier (1998) studied 1064 patients with a variety of malignancies receiving chemotherapy, in whom the mean baseline haemoglobin was > 12 g dl$^{-1}$ in all but two tumour groups. By the third cycle of chemotherapy, nearly two-thirds of patients were anaemic, of whom 28% were mildly anaemic (haemoglobin 10.5 to 12 g dl$^{-1}$), 34% were moderately anaemic (haemoglobin 8 to 10.5 g dl$^{-1}$), and 5% were severely anaemic (haemoglobin < 8 g dl$^{-1}$). In a separate study of 2821 patients receiving cytotoxic chemotherapy, an average of 17% of patients were anaemic (haemoglobin < 11 g dl$^{-1}$) before the first cycle (Dalton et al, 1998). This figure increased to 35% by the

*Correspondence to:* G Demetri sixth cycle. On average, 33% of patients required at least one red blood cell transfusion because of anaemia.

Anaemia after radiotherapy also appears to be common. In a study by Harrison et al (2000), 48% of patients presenting for radiotherapy for solid tumours were anaemic (haemoglobin < 12 g $dl^{-1}$) before treatment; this figure increased to 57% with the completion of therapy.

### IMPACT OF ANAEMIA ON QUALITY OF LIFE

Anaemia can affect many aspects of QOL and can result in functional deficits such as decreased exercise capacity, headaches, dyspnoea, loss of libido, and dizziness (Cella, 1997). This lowers the subjective sense of well-being and impairs other important aspects of patients' lives such as the ability to work, interact socially, and enjoy leisure activities (Yellen et al, 1997).

One of the most pronounced, and most studied, clinical symptoms of anaemia is fatigue (Khayat, 2000). Fatigue is the most prevalent symptom reported by patients with cancer, and has reportedly been regarded by patients as more important than nausea and vomiting (Stone et al, 2000). In a recent survey, approximately three-quarters of patients with cancer receiving chemotherapy (with or without radiotherapy) reported experiencing fatigue (defined as a 'general feeling of debilitating tiredness or loss of energy') (Curt et al, 2000). Fatigue has a large impact on QOL (Cella, 1997), and 91% of patients reported that it prevented them from leading a 'normal' life (Curt et al, 2000). For 61% of patients, fatigue was more significant than cancer-related pain (Vogelzang et al, 1997). Fatigue also affects work schedules, with employed patients using an average of 4.2 sick/vacation days per month, during or immediately after treatment, as a direct result of fatigue (Curt et al, 2000).

Despite the impact on patients' QOL, oncologists are commonly reluctant to treat fatigue (Vogelzang et al, 1997; Curt, 2000). Seventy-seven percent of patients who discussed fatigue with their oncologists reported that they were told that fatigue was something they had to endure, and 40% were told that there was nothing their oncologist could do (Vogelzang et al, 1997). Only 6% of oncologists prescribed medication for fatigue, according to patients (Curt et al, 2000). However, when oncologists were questioned in a separate survey regarding the importance of treating fatigue, 80% believed that fatigue is overlooked or under-treated by their peers (Vogelzang et al, 1997).

In patients with cancer, anaemia and fatigue are closely linked, but this association is not easy to characterize. Just as fatigue is not the only symptom of anaemia, anaemia is not the only cause of fatigue. Although fatigue may be multifactorial, anaemia is an important contributing factor to fatigue in cancer patients and one that can be treated.

An objective correlation exists between haemoglobin values and QOL in patients with cancer (Cella, 1997; Yellen et al, 1997). Cella (1997) developed and tested derivations of the Functional Assessment of Cancer Therapy (FACT) measurement systems that were specifically designed to evaluate the impact of fatigue (FACT-F) or anaemia (FACT-An) on QOL in cancer patients. The results clearly showed that patients with haemoglobin values > 12 g $dl^{-1}$ experienced significantly less fatigue, fewer non-fatigue anaemia symptoms, better physical well-being, better functional well-being, and higher general QOL than those whose haemoglobin was < 12 g $dl^{-1}$. Furthermore, haemoglobin values were shown to be associated with overall QOL, with patients with higher haemoglobin values experiencing a higher QOL.

### BENEFITS OF TREATING ANAEMIA

### Improved quality of life

Numerous studies, both placebo-controlled and open-label, have consistently shown that increasing haemoglobin values has a measurable and significant effect on QOL parameters in cancer patients whether they are receiving (Abels, 1993; Case et al, 1993; Henry and Abels, 1994; Leitgeb et al, 1994; Glaspy et al, 1997; Demetri et al, 1998) or not receiving concurrent chemotherapy (Ludwig et al, 1995). In all of these studies, haemoglobin was increased by the administration of recombinant human erythropoietin (rHuEPO, epoetin alfa).

Large-scale clinical trials designed to study the effect of rHuEPO on QOL in the setting of community oncology practice rather than in the more artificial environment of a phase 3 clinical trial have provided compelling and consistent data showing the importance of treating anaemia in cancer patients (Glaspy et al, 1997; Demetri et al, 1998). In an open-label study, in which rHuEPO was administered to 2342 patients receiving cytotoxic chemotherapy, patients' mean energy levels increased by 38%, activity increased by 32%, and overall QOL increased by 24% after 4 months of therapy, all significant increases ($P$ < 0.001) (Glaspy et al, 1997). A later study involving 2370 patients reported similar results, noting that increased haemoglobin values from baseline were associated with improved activity level, energy, and overall well-being (Figure 1). Furthermore, a direct and statistically significant association was shown by regression analysis between the increase in overall QOL and the increase in haemoglobin level from baseline ($r$ = 0.235; $P$ < 0.001) (Demetri et al, 1998).

The beneficial effect of rHuEPO was shown to be independent of anti-tumour response in a prospective analysis of 2117 patients (Demetri et al, 1998). Patients who had no increase in haemoglobin values did not experience a significant increase in QOL, regardless of whether they achieved a complete or partial response

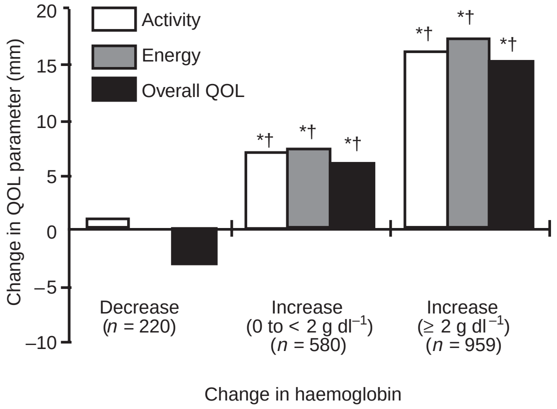

**Figure 1** Change from baseline to final quality of life (QOL) score by haemoglobin change. Activity, energy, and overall QOL were assessed using linear analogue scales and analysed based on corresponding haemoglobin value changes. *Significantly different from baseline ($P$ < 0.01).
† Significantly different from adjacent haemoglobin change group ($P$ < 0.01).
Reproduced with kind permission of Lippincott Williams & Wilkins from Demetri et al (1998)

*© 2001 Cancer Research Campaign*

to chemotherapy, or had stable disease. Direct and statistically significant correlations between haemoglobin value change and change in overall QOL for complete response after chemotherapy ($r = 0.242$; $P < 0.001$), partial response ($r = 0.275$; $P < 0.001$), and stable disease ($r = 0.253$; $P < 0.001$) were shown, but not for progressive disease ($r = 0.084$; $P = 0.072$). Patients with progressive disease experienced some benefits from an increase in haemoglobin; however, the magnitude of the effect in this subset of patients, as one might expect, was far less than in patients whose tumours were controlled by anticancer therapy.

Cleeland et al (1999) analysed the results from the open-label trials by Glaspy et al (1997) and Demetri et al (1998) and showed that the largest improvement in QOL occurred when haemoglobin values increased from 11 to 12 g $dl^{-1}$. This relationship was maintained after controlling for tumour type and status, transfusions, number of days on study, the extent of chemotherapy and radiotherapy, and feelings of pain and/or nausea. This result clearly shows the benefits of even small increases in haemoglobin and demonstrates that a decrease in haemoglobin to only slightly below normal levels is frequently associated with significant reductions in QOL.

### Possibilities for improved treatment outcomes

Many studies have found that inadequate oxygenation at the tumour site and/or low haemoglobin values are associated with poor treatment outcome after curative radiotherapy (Fein et al, 1995; Dubray et al, 1996; Fyles et al, 1998; Grogan et al, 1999). Adequate tumour oxygenation is known to be necessary for an optimal response to radiotherapy (Glaspy and Cavill, 1999), thus it is theoretically possible that decreased haemoglobin (which may contribute to lowered oxygenation at the tumour site) may also have an effect on the success of therapy.

In a study of 74 patients with cervical cancer, disease-free survival was statistically significantly associated with tumour oxygenation ($P = 0.02$) (Fyles et al, 1998). Other studies have shown a statistically significant association between anaemia and reduced local control or survival in cervical cancer or head and neck cancer (Fein et al, 1995; Dubray et al, 1996; Warde et al, 1998; Grogan et al, 1999). Dubray et al (1996) found that even moderate anaemia significantly correlated with a worse treatment outcome in squamous cell carcinoma of the head and neck (Figure 2). However, it is unclear whether low haemoglobin directly contributes to reduced tumour control and survival after radiotherapy, or whether it is merely a marker of advanced disease in patients for whom successful treatment outcomes are less likely, regardless of tumour oxygenation. Thus, randomized, controlled trials are needed to assess the effect of anaemia correction on survival and disease control.

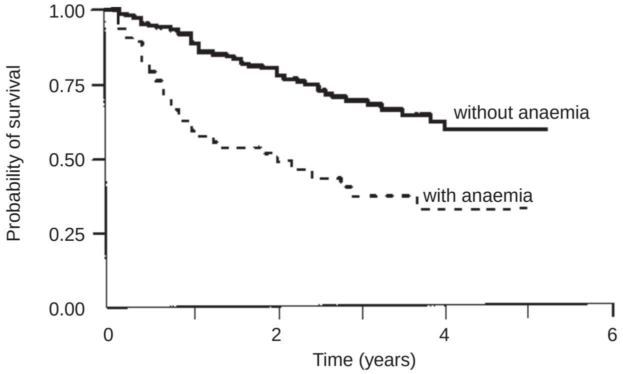

**Figure 2** Actuarial probability of overall survival according to anaemic status. The haemoglobin cut-off level for anaemia was 13.5 g $dl^{-1}$ for men and 12 g $dl^{-1}$ for women. Reproduced with kind permission of the Radiological Society of North America Inc. from Dubray et al (1996)

### ISSUES IN THE CURRENT MANAGEMENT OF ANAEMIA

Anaemia is widely believed to be under-recognized and under-treated (Glaspy et al, 1997; Ludwig, 1999). Most physicians do not administer transfusions until severe anaemia develops (haemoglobin < 8 to 9 g $dl^{-1}$) (Coiffier, 1998; Estrin et al, 1999; Glaspy and Harper, 2000; Throuvalas et al, 2000) and a recent survey of 3472 patients treated by 20 community oncologists found that 52 to 70% of anaemic patients (haematocrit < 30%) undergoing chemotherapy did not receive treatment with rHuEPO (Lawless et al, 2000).

There are several possible explanations why anaemia is under-treated: lack of awareness of the incidence and impact of anaemia; inadequacies in the current treatment options; and the existence of several key uncertainties surrounding the management of anaemic cancer patients.

### Lack of awareness of the incidence and impact of anaemia

Despite the overwhelming evidence supporting the value of treating anaemia, and particularly that described as ‘functional’ anaemia, it appears that many physicians are reluctant to administer therapy. Treatment is usually reserved for patients with severe anaemia, when haemoglobin values decrease to 8 or 9 g $dl^{-1}$ or serious cardiovascular symptoms are apparent (Glaspy and Harper, 2000). This failure to administer therapy until haemoglobin has decreased to such a low level may be attributed, at least in part, to lack of awareness of the benefits of treating anaemia, particularly mild-to-moderate anaemia. A lack of awareness of the high incidence of anaemia accompanying particular malignancies and chemotherapy regimens is also likely to contribute to the low rate of treatment.

### Inadequacies in the current treatment options

*Transfusions*

Red blood cell transfusions are a rapid and reliable method of correcting anaemia, particularly in cases where anaemia is life-threatening. However, many real and perceived risks are associated with the procedure, such as infection, allergic and/or febrile reactions, and the theoretical risk of transfusion-associated immunosuppression (Ludwig and Fritz, 1998a; Goodnough et al, 1999) (Table 1). In addition, there is the inconvenience, to both clinician and patient, of administering the transfusions for only transient benefit. For patients who may already be myelosuppressed and feeling poorly, the risks of allergic and febrile reactions to transfusions are not favourable. Furthermore, patients often express very strong preferences to avoid undergoing transfusion (Table 2).

© 2001 Cancer Research Campaign

**Table 1** Risks of complications from blood transfusions

| Risk factor | Estimated frequency | | No. deaths per million units |
|---|---|---|---|
| | Per million units | Per actual unit | |
| Infection | | | |
| Viral | | | |
| Hepatitis A | 1 | 1/1 000 000 | 0 |
| Hepatitis B | 7–32 | 1/30 000–1/250 000 | 0–0.14 |
| Hepatitis C | 4–36 | 1/30 000–1/150 000 | 0.5–17 |
| HIV | 0.4–5 | 1/200 000–1/2 000 000 | 0.5–5 |
| HTLV types I and II | 0.5–4 | 1/250 000–1/2 000 000 | 0 |
| Parvovirus B19 | 100 | 1/10 000 | 0 |
| Bacterial contamination | | | |
| Red cells | 2 | 1/500 000 | 0.1–0.25 |
| Platelets | 83 | 1/12 000 | 21 |
| Acute haemolytic reactions | 1–4 | 1/250 000–1/1 000 000 | 0.67 |
| Delayed haemolytic reactions | 1000 | 1/1000 | 0.4 |
| Transfusion-related acute lung injury | 200 | 1/5000 | 0.2 |

HIV = human immunodeficiency virus; HTLV = human T-cell lymphotropic virus.
Reproduced with kind permission of the Massachusetts Medical Society from Goodnough et al, 1999. Copyright © 1999 Massachusetts Medical Society. All rights reserved.

**Table 2** Limitations of current therapies for anaemia

| Transfusions | rHuEPO |
|---|---|
| Inconvenient | Effective in only 50–60% of patients |
| Transient benefit | Response can take 4 weeks or longer to occur |
| Patients' preference to avoid | No adequate predictors of response |
| Risk of infections | Administration not necessarily synchronized with chemotherapy |
| Tumour potentiation due to immune suppression (as yet unproven) | Frequent injections require numerous patient visits |
| Haemolytic reactions | Tumour potentiation (as yet unproven) |
| Risk of allergic reactions | |
| Iron overload | |
| Volume expansion | |
| Loss of efficacy in patients with antibodies | |
| Only minor impact on quality of life | |
| Supply is limited | |
| Handling is time-consuming | |

Adapted from Ludwig and Fritz (1998a) and Procrit™ prescribing information (Ortho Biotech, 1999).

### *Recombinant human erythropoietin*

Despite the well-documented efficacy of rHuEPO in increasing haemoglobin and reducing the need for transfusions, this agent is still not used routinely for the management of patients receiving chemotherapy as it is for the support of haemoglobin levels in patients with other chronic diseases such as renal failure. This under-use may be due in part to the limitations of this therapy, including less-than-optimal response rates and the lack of adequate reliable predictors of response (Table 2). In addition, several outstanding issues remain to be clarified, such as the optimal dose and schedule, and when to stop or increase the dose of the drug.

*Inconvenience of rHuEPO* Inconvenience may seem a minor limitation of rHuEPO therapy to some healthcare providers, however, to patients it may be of great importance. Since the original regulatory approval of rHuEPO, it has been recommended that rHuEPO be given 3 times weekly, a schedule that does not always correspond with the administration of chemotherapy. The use of once-weekly dosing, recently reported by Gabrilove et al (1999), may help alleviate this problem and encourage greater patient compliance.

*Suboptimal response rates to rHuEPO* For the 50 to 60% of patients who respond to therapy (variably defined as an increase in haemoglobin ≥ 2 g $dl^{-1}$ or an increase in haematocrit of ≥ 6%), rHuEPO is an effective and well-tolerated treatment, increasing haemoglobin values, reducing the need for transfusions, and improving overall QOL (Abels, 1993; Leitgeb et al, 1994; Glaspy et al, 1997; Demetri et al, 1998). However, therapy is ineffective for the 40 to 50% of patients who do not respond, and the relatively slow time to response may cause some patients and physicians to terminate rHuEPO therapy prematurely due to lack of perceived benefit. In one series of patients, the median time to

 © 2001 Cancer Research Campaign response was approximately 4 weeks, but it may require as long as 12 weeks to determine responsiveness in an individual patient (Ludwig and Fritz, 1998b). Clearly, administering and monitoring response to therapy for up to 3 months for no ultimate gain is not ideal. Almost half of those patients (44%) who fail to respond to the initial dose of rHuEPO will respond to an increased dose (Demetri et al, 1998); however, predicting these responders has not been possible by any prospective indicators.

*Predicting response to rHuEPO* Potentially, the impact of the less-than-optimal response rates can be lowered if physicians are able to predict, before commencement of therapy, which patients are likely to respond and thus direct treatment at these patients. An alternative method is to identify non-responsive patients early in the treatment course and terminate ineffective therapy appropriately.

Many potential predictive factors have been studied, including pre-treatment erythropoietin (EPO) levels (Abels, 1992; Cascinu et al, 1994) and changes in indicators of erythropoietic response measured 2 to 4 weeks after commencement of treatment (Ludwig et al, 1994; Glaspy et al, 1997). However, although numerous variables (haemoglobin, EPO, ferritin, neopterin, C-reactive protein, transferrin receptors, transferrin, serum iron, haematocrit, red blood cells, reticulocytes and $\alpha_1$-antitrypsin), measured after 2 weeks, have been found to significantly correlate with response, none was associated strongly enough to serve as a reliable single prognostic indicator (Ludwig et al, 1994). Of the factors studied, the change in haemoglobin value after 2 weeks proved the most reliable.

Based on these analyses, Ludwig et al (1994) predicted that, if after 2 weeks of therapy the serum EPO level is $\geq$ 100 mU ml$^{-1}$ and haemoglobin value has not increased by at least 0.5 g dl$^{-1}$, the patient is unlikely to respond to rHuEPO (predictive power, 93%); otherwise response may be predicted with an accuracy of 80% (Ludwig et al, 1994). Conversely, if the serum EPO level is < 100 mU ml$^{-1}$ and haemoglobin value has increased by $\geq$ 0.5 g dl$^{-1}$, the patient is very likely to respond (predictive power, 95%).

With the use of increased doses of rHuEPO, however, it may not be prudent to immediately classify patients as unresponsive because they did not achieve an increase in haemoglobin at the standard dose. In the trial by Demetri et al (1998), patients whose haemoglobin increased by < 1 g dl$^{-1}$ received a doubled dose of rHuEPO (to 20 000 U 3 times weekly). Of these, 44% went on to achieve either an increase in haemoglobin of $\geq$ 2 g dl$^{-1}$ or a haemoglobin level $\geq$ 12 g dl$^{-1}$ by the end of the study, demonstrating that initial failure to respond does not necessarily indicate that a patient will not benefit from increased doses of rHuEPO.

Thus, despite much research, highly reliable predictors of response to rHuEPO do not exist, making the targeting of rHuEPO therapy to appropriate patients difficult. Complications such as infections and functional iron deficiency, which can impair response to rHuEPO, may further confound the problem (Beguin, 1998).

*Cost* Red blood cell transfusions have historically been viewed as less expensive than rHuEPO (Ludwig and Fritz, 1998a; Mercadante et al, 2000). It is difficult, however, to gain a current, accurate measurement of the cost of acquiring, handling, processing, storing and administering blood; the costs associated with the complications of transfusions; and the indirect economic costs to patients due to travelling to a transfusion centre and/or absence from work. One analysis placed the cost of collecting, testing, and administering blood, and treating complications at £192 (US$324) per transfusion (Dalton et al, 1998). A more recent retrospective study of 517 patients with haematologic or solid tumours estimated the cost of a two-unit transfusion to be US$938, but did not take into consideration the cost of treating complications (Cremieux et al, 2000). As the combination of fewer donations and increased demand decreases the supply of available blood and the number of sophisticated screening tests rises, transfusion costs are likely to increase.

Pharmacoeconomic analyses are therefore needed that incorporate (a) the cost of transfusions (handling and administering blood, and managing complications); (b) the economic consequences of lost productivity associated with administering transfusions; (c) the cost of administering rHuEPO once the optimal dose and schedule is identified; and (d) the cost of administering rHuEPO once physicians are able to target therapy to those patients most likely to respond and/or terminate therapy once a response is ruled out. Furthermore, the economic costs of lost productivity by patients who are unable to work due to symptomatic anaemia must be considered, and the psychological costs of patient preferences to avoid transfusions should also be taken into account.

*Outstanding issues* Further studies using rHuEPO are needed to address additional issues such as the optimal dose and schedule (Glaspy et al, 1997), when to stop the drug because of lack of response, and when to increase the dose (Mercadante et al, 2000). It has been suggested that an initial dose of rHuEPO of 150 U kg$^{-1}$ is given three times weekly subcutaneously, which is increased to 300 U kg$^{-1}$ if the haemoglobin value does not increase by $\geq$ 1 g dl$^{-1}$ after 4 weeks of therapy. A patient who does not respond to the doubled dose is unlikely to respond to higher doses (Glaspy et al, 1997) and should therefore have therapy terminated at this point. However, whether this treatment pattern is followed in routine clinical practice or results in the most efficient and effective use of the agent is unclear.

### Key uncertainties surrounding the management of anaemic cancer patients

Monitoring, identifying and effectively treating anaemic cancer patients is not an easy task. Physicians must consider many issues on a regular basis to ensure that their patients do not develop, and subsequently suffer, the effects of anaemia.

An issue that is currently unclear is exactly how to determine an appropriate trigger point for therapeutic intervention. That is, if a patient's haemoglobin value is decreasing, which criteria should be used to determine the point at which the patient requires therapy? Clearly, physical symptoms should be considered as well as haemoglobin value. The study by Cleeland et al (1999) showed that the greatest improvements in QOL occur when haemoglobin values increase from 11 to 12 g dl$^{-1}$. This suggests that a haemoglobin value at or below 11 g dl$^{-1}$ may be an appropriate trigger point in those patients whose symptoms have not already necessitated intervention. However, as some patients experience the effects of anaemia before their haemoglobin decreases below 11 g dl$^{-1}$, it seems unlikely that using a standard haemoglobin value as a trigger point will identify all patients who could benefit from therapy with an erythropoietic agent.

Thus, it is prudent to consider both symptoms and haemoglobin values before making treatment decisions; however, the relative value placed on each measurement remains to be defined.

© 2001 Cancer Research Campaign 

Whatever the outcome, patients should not have to wait until their anaemia becomes debilitating to receive treatment. If anaemic patients can be identified and treated while their haemoglobin values are decreasing, and before the symptoms of anaemia appear, therapy can be initiated before severe anaemia develops.

As studies show that many, but not all, cancer patients experience anaemia (Coiffier, 1998; Dalton et al, 1998; Groopman and Itri, 1999; Harrison et al, 2000), it would be of great benefit to be able to predict whether a patient is at high risk of developing anaemia and/or has a low tolerance to the complications of anaemia and blood transfusions. Such knowledge would assist physicians in monitoring patients more closely and/or administering therapy with an erythropoietic agent before anaemia becomes symptomatic. A recent study by Ray-Coquard et al (1999) may assist in this goal; however, it requires validation in a large, multicentre study.

Finally, there are no universally accepted guidelines addressing the most effective methods of monitoring cancer patients for anaemia, and once identified, managing the condition. The development of such guidelines may assist in improving current treatment practices and result in more widespread and effective management of anaemic patients with cancer.

### THE FUTURE

A recent addition to the family of growth factors is darbepoetin alfa (ARANESP™, Amgen Inc, Thousand Oaks, CA). Darbepoetin alfa is a novel erythropoiesis stimulating protein (NESP), and represents a new generation of erythropoiesis stimulating proteins. A license application has been filed worldwide for its use to treat renal anaemia, and it is under further development in the oncology-haematology setting.

Experimental evidence demonstrating a direct relationship between the amount of sialic acid-containing carbohydrate side chains on rHuEPO, and serum half-life and in vivo biological activity prompted the development of NESP. This protein has an increased sialic acid content and thus, an approximately 3-fold longer serum half-life than rHuEPO and increased biological activity (Egrie et al, 1997; Macdougall et al, 1999). NESP binds to EPO receptors and stimulates erythropoiesis (Macdougall, 2000). In animal studies, NESP was approximately 3.6 times as potent as rHuEPO in increasing the haematocrit in normal mice when injected three-times weekly and was 20-fold more efficacious than rHuEPO when both agents were given in a once-weekly dosing schedule (Egrie et al, 1997).

NESP has undergone clinical testing in more than 2000 oncology and chronic renal failure patients. The agent was well tolerated in clinical trials, with no evidence of antibody formation (Vanrenterghem et al, 1999; Glaspy et al, 2000; Kotasek et al, 2000; Macdougall, 2000; Tseng et al, 2000; Glaspy et al, 2001; Heatherington et al, 2001; Smith et al, 2001). Pharmacokinetic studies in patients with renal failure demonstrated that NESP has a serum half-life of approximately 50 hours after subcutaneous administration (Macdougall et al, 1999). Large clinical trials demonstrated that NESP is effective in the treatment of renal anaemia and that once-weekly and once every second week dosing with the agent is possible, using both the intravenous and subcutaneous routes of administration (Macdougall, 1998; Vanrenterghem et al, 1999).

Following the success of NESP in treating renal anaemia, trials are currently underway (the preliminary results from three of which are included in this supplement) to assess the value of the agent in treating anaemia of cancer (Glaspy et al, 2001; Heatherington et al, 2001; Smith et al, 2001). The dose-escalation study involving 107 patients with solid tumours conducted by Glaspy et al (2001), in which the agent was administered once-weekly to patients receiving chemotherapy, demonstrates a dose–response relationship between NESP and haemoglobin increase. Preliminary data from the dose-escalation study by Smith et al (2001) indicate that NESP is also effective in patients not receiving chemotherapy. At a dose of 2.25 mcg $kg^{-1}$ $wk^{-1}$ of NESP, 72% of patients corrected their haemoglobin values (haemoglobin ≥ 12 g $dl^{-1}$). Time to response was shorter in those patients receiving higher doses of NESP. Heatherington et al (2001) confirmed an extended half-life of NESP in patients with cancer, supporting the possibility that this therapy can be administered less frequently. In fact, a phase 1–2 trial has demonstrated that administration of NESP once every 3 weeks may be clinically effective (Kotasek et al, 2000).

Thus, the data so far show that NESP, through its longer half-life, increased biological activity and less frequent dosing, has great potential to improve the management of anaemia in patients with cancer. Less frequent dosing will allow administration to be more easily synchronized with chemotherapy, and may reduce the compliance burden on the patient and health care resources.

### CONCLUSIONS

Anaemia and fatigue are common among cancer patients and have a measurable impact on QOL. However, anaemia, and particularly mild-to-moderate anaemia, appears under-recognized and under-treated in the practice of oncology. There are several possible reasons for this, some of which can be overcome by increased awareness of the problem posed by anaemia and the clarification of key management issues. Many of the current limitations of rHuEPO may be overcome by the introduction of NESP into routine clinical practice. The data reported so far are encouraging, and reduced dosing frequency, with the potential for more rapid responses, should translate to a reduced burden on both patients and health care resources. Further trials will determine the full potential of NESP in the management of cancer-related anaemia; in the meantime, physicians must remain alert to the development of anaemia in their patients and ensure that patients are managed as effectively as possible.

### REFERENCES

Abels R (1993) Erythropoietin for anaemia in cancer patients. *Eur J Cancer* **29A**: S2–8

Abels RI (1992) Use of recombinant human erythropoietin in the treatment of anemia in patients who have cancer. *Semin Oncol* **19**: 29–35

Beguin Y (1996) Erythropoietin and the anemia of cancer. *Acta Clin Belg* **51**: 36–52

Beguin Y (1998) Prediction of response to optimize outcome of treatment with erythropoietin. *Semin Oncol* **25**: 27–34

Cascinu S, Fedeli A, Del Ferro E, Luzi Fedeli S and Catalano G (1994) Recombinant human erythropoietin treatment in cisplatin-associated anemia: a randomized, double-blind trial with placebo. *J Clin Oncol* **12**: 1058–1062

Case DC, Jr., Bukowski RM, Carey RW, Fishkin EH, Henry DH, Jacobson RJ, Jones SE, Keller AM, Kugler JW and Nichols CR (1993) Recombinant human erythropoietin therapy for anemic cancer patients on combination chemotherapy. *J Natl Cancer Inst* **85**: 801–806

Cella D (1997) The Functional Assessment of Cancer Therapy-Anemia (FACT-An) Scale: a new tool for the assessment of outcomes in cancer anemia and fatigue. *Semin Hematol* **34**: 13–19

Cleeland CS, Demetri GD, Glaspy J, Cella DF, Portenoy RK, Cremieux PY and Itri L (1999) Identifying hemoglobin level for optimal quality of life: results of an incremental analysis. *Proc Am Soc Clin Oncol* **18**: 574a (abstract 2215)

© 2001 Cancer Research Campaign

Coiffier B (1998) Retrospective analysis of hematological parameters and transfusion requirements in non-platinum chemotherapy-treated patients. *Proc Am Soc Clin Oncol* **17**: 90a (abstract 346)

Cremieux PY, Barrett B, Anderson K and Slavin MB (2000) Cost of outpatient blood transfusion in cancer patients. *J Clin Oncol* **18**: 2755–2761

Curt GA (2000) The impact of fatigue on patients with cancer: Overview of FATIGUE 1 and 2. *Oncologist* **5 Suppl 2**: 9–12

Curt GA, Breitbart W, Cella D, Groopman JE, Horning SJ, Itri LM, Johnson DH, Miaskowski C, Scherr SL, Portenoy RK and Vogelzang NJ (2000) Impact of cancer-related fatigue on the lives of patients: new findings from the Fatigue Coalition. *The Oncologist* **5**: 353–360

Dalton JD, Bailey NP, Barrett-Lee PJ and O'Brien MER (1998) Multicenter UK audit of anemia in patients receiving cytotoxic chemotherapy. *Proc Am Soc Clin Oncol* **17**: 418a (abstract 1611)

Demetri GD, Kris M, Wade J, Degos L and Cella D (1998) for the Procrit Study Group. Quality-of-life benefit in chemotherapy patients treated with epoetin alfa is independent of disease response or tumor type: results from a prospective community oncology study. *J Clin Oncol* **16**: 3412–3425

Dubray B, Mosseri V, Brunin F, Jaulerry C, Poncet P, Rodriguez J, Brugere J, Point D, Giraud P and Cosset JM (1996) Anemia is associated with lower local-regional control and survival after radiation therapy for head and neck cancer: a prospective study. *Radiology* **201**: 553–8

Egrie JC, Dwyer E, Lykos M, Hitz A and Browne JK (1997) Novel erythropoiesis stimulating protein (NESP) has a longer serum half-life and greater in vivo biological activity than recombinant human erythropoietin (rHuEPO). *Blood* **90**: 56a (abstract 243)

Estrin JT, Schocket L, Kregenow R and Henry DH (1999) A retrospective review of blood transfusions in cancer patients with anemia. *The Oncologist* **4**: 318–324

Fein DA, Lee WR, Hanlon AL, Ridge JA, Langer CJ, Curran WJ, Jr. and Coia LR (1995) Pretreatment hemoglobin level influences local control and survival of T1-T2 squamous cell carcinomas of the glottic larynx. *J Clin Oncol* **13**: 2077–2083

Fyles AW, Milosevic M, Wong R, Kavanagh MC, Pintilie M, Sun A, Chapman W, Levin W, Manchul L, Keane TJ and Hill RP (1998) Oxygenation predicts radiation response and survival in patients with cervix cancer. *Radiother Oncol* **48**: 149–156

Glaspy J, Bukowski R, Steinberg D, Taylor C, Tchekmedyian S and Vadhan-Raj S (1997) Impact of therapy with epoetin alfa on clinical outcomes in patients with nonmyeloid malignancies during cancer chemotherapy in community oncology practice. *J Clin Oncol* **15**: 1218–34

Glaspy J and Cavill I (1999) Role of iron in optimizing responses of anemic cancer patients to erythropoietin. *Oncology* **13**: 461–473

Glaspy J and Harper P (2000) Discussion. *Semin Oncol* **27**: 16–17

Glaspy J, Meza L, Smith R, Fleishman A, Mendes E and Colowick A (2000) Open-label, phase I/II dose escalation study of ARANESP in patients with chronic anemia of cancer. *Blood* **96**: 154b (abstract 4370)

Glaspy J, Jadeja JS, Justice G, Kessler J, Richards D, Schwartzberg L, Rigas J, Kuter D, Harmon D, Prow D, Demetri G, Gordon D, Arseneau J, Saven A, Hynes H, Boccia R, O'Byrne J and Colowick AB (2001) A dose-finding and safety study of novel erythropoiesis stimulating protein (NESP) for the treatment of anaemia in patients receiving multicycle chemotherapy. *Br J Cancer* **84** (Supp 1): 17–23

Goodnough LT, Brecher ME, Kanter MH and AuBuchon JP (1999) Transfusion medicine. First of two parts: blood transfusion. *N Engl J Med* **340**: 438–447

Grogan M, Thomas GM, Melamed I, Wong FLW, Pearcey RG, Joseph PK, Portelance L, Crook J and Jones KD (1999) The importance of hemoglobin levels during radiotherapy for carcinoma of the cervix. *Cancer* **86**: 1528–1536

Groopman JE and Itri LM (1999) Chemotherapy-induced anemia in adults: incidence and treatment. *J Natl Cancer Inst* **91**: 1616–1634

Harrison LB, Shasha D, White C and Ramdeen B (2000) Radiotherapy-associated anemia: the scope of the problem. *Oncologist* **5**: 1–7

Heatherington AC, Schuller J and Mercer AJ (2001) Pharmacokinetics of novel erythropoiesis stimulating protein (NESP) in cancer patients: preliminary report. *Br J Cancer* **84** (Supp 1): 11–16

Henry DH and Abels RI (1994) Recombinant human erythropoietin in the treatment of cancer and chemotherapy-induced anemia: results of double-blind and open-label follow-up studies. *Semin Oncol* **21**: 21–28

Khayat D (2000) Is anemia a problem for European cancer patients and treating oncologists? *Semin Oncol* **27**: 9–11

Kotasek D, Berg R, Poulsen E and Colowick A (2000) Randomized, double-blind, placebo controlled, phase I/II dose finding study of ARANESP administered once every three weeks in solid tumor patients. *Blood* **96**: 294a (abstract 1268)

Lawless G, Wilson-Royalty M and Meyers J (2000) Epoetin alfa practice pattern usage in community practice sites. *Blood* **96**: 390b (abstract 5446)

Leitgeb C, Pecherstorfer M, Fritz E and Ludwig H (1994) Quality of life in chronic anemia of cancer during treatment with recombinant human erythropoietin. *Cancer* **73**: 2535–2542

Ludwig H (1999) Epoetin in cancer-related anaemia. *Nephrol Dial Transplant* **14**: 85–92

Ludwig H and Fritz E (1998a) Anemia in cancer patients. *Semin Oncol* **25**: 2–6

Ludwig H and Fritz E (1998b) Anemia in cancer patients: patient selection and patient stratification for epoetin treatment. *Semin Oncol* **25**: 35–38

Ludwig H, Fritz E, Leitgeb C, Pecherstorfer M, Samonigg H and Schuster J (1994) Prediction of response to erythropoietin treatment in chronic anemia of cancer. *Blood* **84**: 1056–63

Ludwig H, Sundal E, Pecherstorfer M, Leitgeb C, Bauernhofer T, Beinhauer A, Samonigg H, Kappeler AW and Fritz E (1995) Recombinant human erythropoietin for the correction of cancer associated anemia with and without concomitant cytotoxic chemotherapy. *Cancer* **76**: 2319–2329

Macdougall IC (1998) Novel erythropoiesis stimulating protein (NESP) for the treatment of renal anaemia. *J Am Soc Nephrol* **9**: 258a–259a (abstract A1317)

Macdougall IC (2000) Novel erythropoiesis stimulating protein. *Semin Nephrol* **20**: 375–381

Macdougall IC, Gray SJ, Elston O, Breen C, Jenkins B, Browne J and Egrie J (1999) Pharmacokinetics of novel erythropoiesis stimulating protein compared with epoetin alfa in dialysis patients. *J Am Soc Nephrol* **10**: 2392–2395

Mercadante S, Gebbia V, Marrazzo A and Filosto S (2000) Anaemia in cancer: pathophysiology and treatment. *Cancer Treat Rev* **26**: 303–311

Ortho Biotech (1999) Epoetin alfa prescribing information: Raritan: New Jersey

Ray-Coquard I, Le Cesne A, Rubio MT, Mermet J, Maugard C, Ravaud A, Chevreau C, Sebban C, Bachelot T, Biron P and Blay JY (1999) Risk model for severe anemia requiring red blood cell transformation after cytotoxic conventional chemotherapy regimens. The Elypse 1 Study Group. *J Clin Oncol* **17**: 2840–2846

Smith RE Jr, Jaiyesimi IA, Meza LA, Tchekmedyian NS, Chan D, Griffith H, Brosman S, Bukowski R, Murdock M, Rarick M, Saven A, Colowick AB, Fleishman A, Gayko U and Glaspy J (2001) Novel erythropoiesis stimulating protein (NESP) for the treatment of anaemia of chronic disease associated with cancer. *Br J Cancer* **84** (Supp 1): 24–30

Stone P, Richardson A, Ream E, Smith AG, Kerr DJ and Kearney N (2000) Cancer-related fatigue: inevitable, unimportant and untreatable? Results of a multicentre patient survey. Cancer Fatigue Forum. *Ann Oncol* **11**: 971–5

Throuvalas NA, Antonadou D, Boufi M, Lavey R and Malamos N (2000) Erythropoietin decreases transfusion requirements during radiochemotherapy. *Proc Am Soc Clin Oncol* **19**: 394a (abstract 1558)

Tseng L, Schuller J, Mercer J and Colowick A (2000) The pharmacokinetics of ARANESP™ in oncology patients undergoing multicycle chemotherapy. *Blood* **96**: 156b (abstract 4379)

Vanrenterghem Y, Barany P and Mann J (1999) Novel erythropoiesis stimulating protein (NESP) maintains hemoglobin in ESRD patients when administered once weekly or once every other week. *J Am Soc Nephrol* **10**: 270A (abstract A1365)

Vogelzang NJ, Breitbart W, Cella D, Curt GA, Groopman JE, Horning SJ, Itri LM, Johnson DH, Scherr SL, Portenoy RK and Coalition TF (1997) Patient, caregiver, and oncologist perceptions of cancer-related fatigue: results of a tripart assessment survey. *Semin Hematol* **34**: 4–12

Warde P, O'Sullivan B, Bristow RG, Panzarella T, Keane TJ, Gullane PJ, Witterick IP, Payne D, Liu FF, McLean M, Waldron J and Cummings BJ (1998) T1/T2 glottic cancer managed by external beam radiotherapy: the influence of pretreatment hemoglobin on local control. *Int J Radiat Oncol Biol Phys* **41**: 347–353

Yellen SB, Cella DF, Webster K, Blendowski C and Kaplan E (1997) Measuring fatigue and other anemia-related symptoms with the Functional Assessment of Cancer Therapy (FACT) measurement system. *J Pain Symptom Manage* **13**: 63–74

*© 2001 Cancer Research Campaign* 
